# Surgical management of pulmonary inflammatory pseudotumors: A single center experience

**DOI:** 10.1186/1749-8090-6-18

**Published:** 2011-02-23

**Authors:** Baldassare Mondello, Salvatore Lentini, Mario Barone, Pietro Barresi, Francesco Monaco, Dario Familiari, Annunziata La Rocca, Michele Sibilio, Ignazio Eduardo Acri, Antonio David, Maurizio Monaco

**Affiliations:** 1 Thoracic Surgery Unit, Cardiovascular and Thoracic Department, Policlinic University Hospital, University of Messina, Italy

## Abstract

**Background:**

The pulmonary inflammatory pseudotumor (PIP) is a rare disease. It is still debated whether it represents an inflammatory lesion characterized by uncontrolled cell growth or a true neoplasm. PIP is characterized by a cellular polymorphism.

**Methods:**

We retrospectively analyzed 8 patients with PIP treated by surgery between 2001 and 2009. Preoperative thoracic computed tomography (CT) scan was performed in all cases. All patients underwent preoperative bronchoscopy with washing and brushing and/or transbronchial biopsy and preoperative cytology examination

**Results:**

There were 5 men and 3 women, aged between 38 and 69 years (mean of 58 years). 3 patients (37%) were asymptomatic. The others had symptoms characterized by chest pain, shortness of breath and persistent cough or hemoptysis. 5 patients had neutrophilic leucocytosis. CT scan demonstrated solitary nodules (maximum diameter <3 cm) in 5 patients (62%) and lung masses (maximum diameter >3 cm) in 3 patients (37%). In 2 patients there were signs of pleural infiltration. Distant lesions were excluded in all cases. A preoperative histology examination failed to reach a definitive diagnosis in all patients. At surgery, we performed two lobectomies, one segmentectomy and five wedge resections, these being performed with videothoracoscopy (VATS), except for one patient where open surgery was used. Complete tumor resection was obtained in all patients. According to the Matsubara classification, there were 2 cases of organizing pneumonia, 5 cases of fibrous histiocytoma and one case of lymphoplasmacytoma. All patients were discharged alive from hospital between 4 and 7 days after surgery. At follow-up CT scan performed annually (range 11 to 112 months) (mean 58 months), there were no residual lesions, neither local nor distant recurrences.

**Conclusions:**

PIP is a rare disease. Many synonyms have been used for this disease, usually in relation to the most represented cell type. The true incidence is unclear. Preoperative diagnosis is difficult to reach, despite performing a bronchoscopy or a transparietal needle aspiration. Different classifications have been proposed for PIP. Either medical, radiation or surgical therapy has been used for PIP. Whenever possible, surgery should be considered the standard treatment. Complete surgical resection is advocated to prevent recurrence.

## Background

Pulmonary inflammatory pseudotumor (PIP) is a rare disease and it is still debated whether it represents an inflammatory lesion characterized by uncontrolled cell growth or a true neoplasm, as recently suggested [1,2]. PIP is characterized by a cellular polymorphism, but trans-bronchial and trans-thoracic biopsies are often inconclusive for diagnosis [3]. Surgery is often useful for both treatment and diagnosis [4]. Complete resection is considered essential to prevent relapses [5].

In this study, the authors report their experience on the management of patients presenting with PIP.

## Methods

Data prospectively entered into the registry of our surgical thoracic unit were analyzed. We retrospectively analyzed 8 patients with PIP treated by surgery between 2001 and 2009.

Preoperative symptoms, concomitant disease and abnormal blood test results for all patients in the study group were recorded.

Preoperative thoracic computed tomography (CT) scans were performed in all cases and extended to the abdomen and skull. In the three most recently treated patients, a Fluorodeoxyglucose (^18^F) Positron emission tomography (FDG-PET) scan was performed as well.

All patients underwent preoperative bronchoscopy with washing and brushing and/or transbronchial biopsy and preoperative cytology examination. All patients underwent surgery either by thoracotomy or by video assisted thoracoscopy (VATS) after lung function study. An intraoperative frozen section histology study was performed in all cases.

### Histology study

For a definitive histology study the following procedures were used. The surgical specimens were fixed in 10% formalin solution, embedded in paraffin, cut into sections of 4 μm, stained with hematoxylin and eosin and then subjected to conventional histology.

For immunohistochemical techniques we used antibodies against vimentin, cytokeratins, desmin, smooth muscle actin and epithelial membrane antigens.

Ultrastructural study by electron microscopy was performed after histology sections were fixed with 2.5% glutaraldehyde solution, post-fixed with osmic tetroxide and embedded in an epoxidic resin.

Patients were regularly seen at our out-patient clinic for postoperative follow-up with CT scan performed annually to rule out recurrences.

## Results

Between January 2001 and December 2009, we treated 8 patients affected by PIP, 5 men and 3 women, aged between 38 and 69 years (mean of 58 years).

Three patients (37%) were asymptomatic and lung nodules on radiological examination were occasionally detected. 5 patients were admitted to our surgical unit for symptoms characterized by chest pain, shortness of breath and persistent cough or hemoptysis, despite antibiotic and anti-inflammatory therapy.

Three patients had concomitant diseases at the time of hospital presentation: One had arterial hypertension, one had chronic obstructive pulmonary disease and one had viral hepatitis. This last patient had also had previous heart surgery for endocarditis. A fourth patient had a previous history of surgery for ovarian cancer.

Blood tests performed on admission were normal in 3 patients. The remaining 5 patients had neutrophilic leucocytosis without other non-specific signs of inflammation.

**Figure 1:**
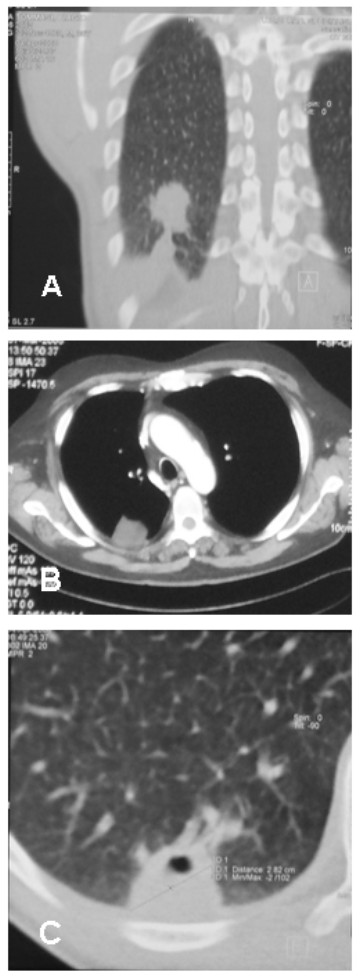
**Computed tomography in patients with pulmonary inflammatory pseudotumor**: A) Non-calcified right lower lobe lung tumor with irregular margins and pleural bridging. B) Non-calcified right lower lobe lung tumor with adjacent pleural thickening. C) Tumor with partial internal cavitation and pleural infiltration.

Computed tomography (CT) scan demonstrated solitary nodules (maximum diameter <3 cm) in 5 patients (62%) and lung masses (maximum diameter >3 cm) in 3 patients (37%). In 6 patients the CT scan showed findings of parenchymal tumors without signs of infiltration; in the other 2 patients there were signs of pleural infiltration (Figure 1). Distant lesions were excluded in all cases.

The 3 patients who underwent FDG-PET scan had a focus of activity with SUV (Standardized Uptake Value) values between 6.2 and 9.8.

Preoperative bronchoscopy was negative in 7 patients. In 1 patient we found bleeding from the right basal pyramid and bronchial brushing cytology was falsely positive for carcinoma with a finding of atypical epithelial cells, lymphocytes and histiocytes. In 3 patients with peripheral nodules we performed US-guided percutaneous needle aspiration. A preoperative histology examination failed to reach a definitive diagnosis in all patients.

At surgery, we performed two lobectomies, one segmentectomy and five wedge resections, these being performed with videothoracoscopy (VATS), except for one patient where open surgery was used. Complete tumor resection was obtained in all patients. The maximum tumor diameter was between 2.5 and 5 cm, with the gross appearance of a well circumscribed mass without a fibrous capsule. Microscopic results were characterized by a collection of inflammatory mesenchymal cells (histiocytes, plasma cells, lymphocytes and spindle cells). Intrapulmonary and mediastinal lymph nodes were found in all cases free from invasion.

**Figure 2:**
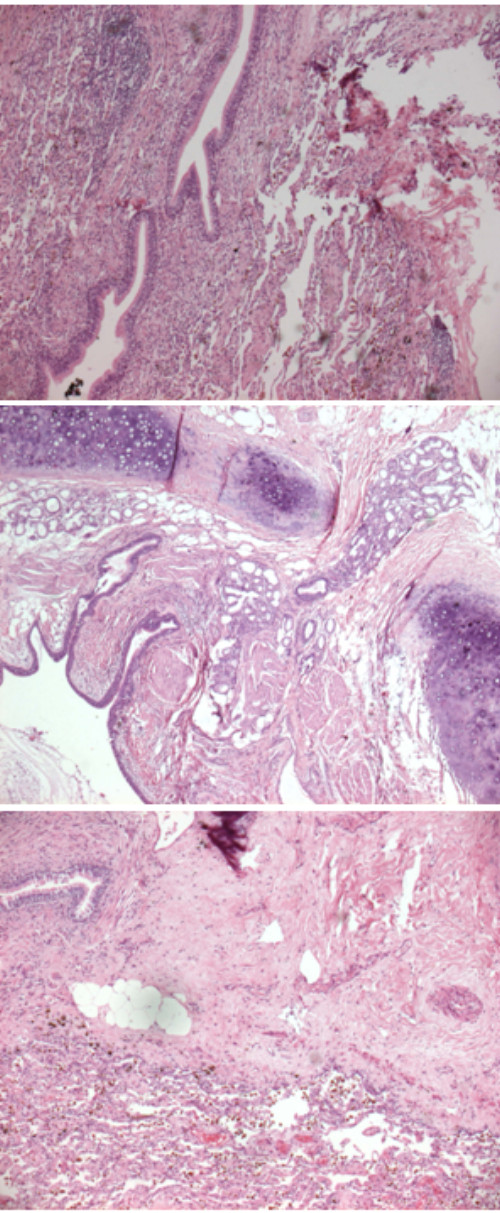
**Histology study at low and high magnification of lung pseudotumor: "organizing pneumonia" type following Matzubara classification**. There are areas of necrosis with inflammatory infiltration of macrophages and lymphocytes.

**Figure 3:**
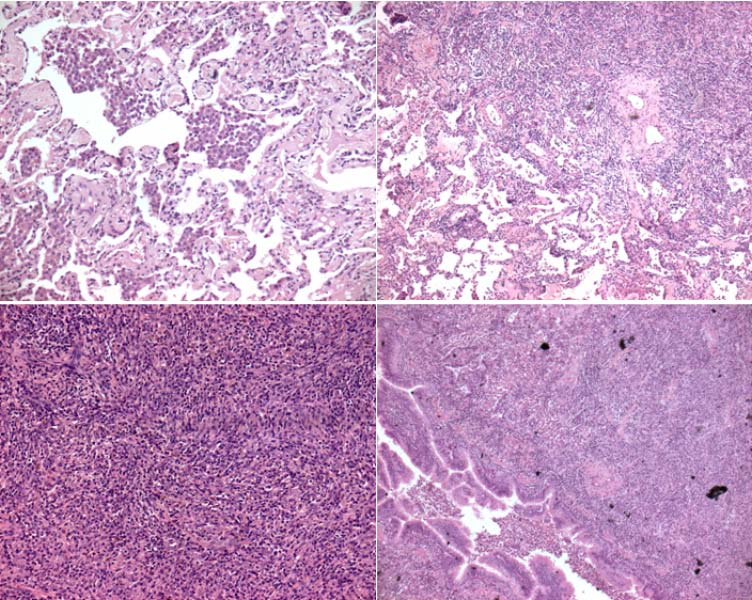
**Histology study at low and high magnification of lung pseudotumor: "fibrous histiocytoma" type following Matzubara classification**. There is a nodular area with large amount of histiocytes, lymphocytes, plasma cells and ialin fibrous connective tissue.

According to the Matsubara classification, microscopic examination revealed 2 cases of organizing pneumonia (Figure 2), 5 cases of fibrous histiocytoma (Figure 3) and one case of lymphoplasmacytoma.

The postoperative course was uneventful in all cases. The length of hospital stay was between 4 and 7 days.

At follow-up CT scan performed annually (range 11 to 112 months) (mean 58 months), there were no residual lesions, neither local nor distant recurrences One patient died of unrelated disease 23 months after surgery. Two patients escaped from follow-up.

## Discussion

Although inflammatory pseudotumors may develop in different organs, such as brain and liver, the lung is the preferred site [1,4]. PIP is a rare disease with a reported incidence between 0.04 to 1.2% of all lung cancers [4]. Many synonyms have been used for this disease, usually in relation to the most represented cell type: plasma cell granuloma, inflammatory myofibroblastic tumor, fibroxantoma, histiocytoma, or pseudoneoplastic pneumonia [4]. The true incidence is unclear, as well as the clinical history in some cases and the response to different therapies [4]. Still nowadays we discuss the nature of this lesion: inflammatory or neoplastic [6]. According to some authors PIP represents a non-neoplastic process characterized by the uncontrolled growth of inflammatory cells [4]. The exact etiology of this inflammatory reaction is unknown and many hypotheses have been raised. The hypothesis of an immune disorder seems to prevail: i.e., a response to viral infection such as to human herpes virus 8 or an antigen-antibody reaction [7]. According to others, PIP represents a true neoplasm, benign or of low-grade malignancy, in consideration of the slow and localized growth [8,9]. This opinion is supported by the detection of cases of PIP with local aggressiveness and infiltration of pulmonary vessels, heart, chest wall, vertebrae and diaphragm, or by detection of cases with distant metastasis or multicentre disease [4,10-12]. Recently discovered cytogenetic abnormalities on chromosome 2p23 would support a neoplastic etiology for this disease [13-15]. PIP is more common in young adults and does not show sex predilection [9].

Patients may remain asymptomatic in 30 to 70% of cases, the disease being occasionally detected on chest radiological examination performed for other reasons [3]. When symptoms occur they are represented by cough, fever, hemoptysis, weight loss, chest pain and respiratory infections due to endobronchial growth or mediastinal invasion [3].

There are no specific radiological signs for PIP. Radiological examination may show the appearance of solitary nodules or masses that may present as either calcified and well demarcated, with no evidence of malignancy, or with irregular contours [16]. Computed tomography (CT) usually shows single nodules or single masses, and multiple locations in only 5% of cases [17]. Agrons et al. reported signs of hilar, mediastinal or bronchial infiltration in 16% of the examined cases [17].

Fluorodeoxyglucose (^18^F) Positron emission tomography (FDG-PET) scan shows an uptake similar to that of malignant tumors [18]. We believe the use of FDG-PET scan is also useful for the study of mediastinal lymph nodes.

As often seen in previously reported series, also in our experience we did not reach a preoperative diagnosis with certainty, despite performing a bronchoscopy in all cases and a transparietal needle aspiration in 3 cases.

Frozen sections from transbronchial or transparietal biopsy are often difficult to interpret, giving an uncertain diagnosis [3,6]. Due to the large number of inflammatory cells and fibroblast proliferation, differential diagnosis would include conditions such as fibrohistiocystic neoplasm, plasmocytomas, Hodgkin's sclerosing lymphoma, primary lung cancer or sarcoma, or mediastinal fibrosis [3,6,19]. However, frozen sections are usually able to rule out malignancies [3,6]. For the reasons mentioned, surgery would be recommended not only as treatment but also to reach a definitive diagnosis [4,20,21].

Different classifications have been proposed for PIP.

According to Cerfolio, PIP is histologically classified into two types with respect to its local invasiveness. The first type, called non-invasive PIP, mostly presenting in asymptomatic patients, appears as a small lesion without invasiveness of blood vessels or adjacent structures and usually easily resectable with a wedge resection [4]. The second type of Cerfolio classification is called invasive PIP and is usually diagnosed in younger patients, with symptoms such as fever, fatigue and weight loss. In these cases, the PIP usually has a larger size and may present with chest wall or mediastinal invasion, requiring lobectomy or pneumonectomy for a complete surgical resection. Invasive PIP may macroscopically appear as lesions infiltrating tissue planes and histologically characterized by nuclear atypia and frequent mitosis [4].

Matsubara et al, reporting on their experience, distinguish three subtypes of PIP according to clinicopathological characteristics: 1) organizing pneumonia type (44%), fibrous histiocytoma type (44%), and lymphoplasmacytic type (12%) [6]. These authors with their classification consider most likely an inflammatory genesis for PIP [6].

Colby et al instead classify PIP into a fibrohistiocytic subtype and a plasma cell granuloma subtype [22].

A recent classification of the World Health Association (WHA) classifies the PIP into three main histologic patterns: 1) myxoid vascular, 2) compact cord cell and 3) hypocellular fibrous. The three different patterns may coexist in the same lesion [23].

Neither the Matsubara nor the WHA classification seems to have a prognostic value [5].

Either medical, radiation or surgical therapy has been used for PIP.

Corticosteroid therapy has been proposed in the case of inoperable patients, for concurrent cardio-respiratory diseases, for unresectable lesions or in case of recurrences [24,25]. The reported results are extremely variable, ranging from ineffectiveness to complete disease regression [25,26].

Radiation therapy is usually reserved for cases of aggressive PIP, or after incomplete excision, or for postoperative recurrence or for patients at high surgical risk [27,28]. The alternative roles of radiation therapy or chemotherapy versus surgery is controversial [3,4,21,28]. It is a shared opinion that whenever possible, surgery should be considered the standard treatment [2]. Even in recurrence, whenever possible, surgical resection is advocated, allowing even in these cases a longer disease-free interval [4].

Complete surgical resection is advocated to prevent recurrence. Prognosis after surgical radical resection is usually excellent [4,9,21]. Long-term follow-up is still required due to the possibility of local or distant recurrence, even after several years [4,24].

Whenever possible, wedge resection should be considered the first line treatment. It would allow saving of lung parenchyma and intraoperative histological examination to exclude malignancy [2,5]. If needed, lobectomy or pneumonectomy would be performed to ensure radical resection, or if diagnosis of malignancy may not excluded. En bloc resection may be needed in cases of chest wall invasion or main bronchus, pericardium or diaphragm involvement [2].

A collection of case studies reported a survival rate at 5 and 10 years, respectively, of 91 and 77%, with values similar to those of a low-grade malignant neoplasm [29].

We should also mention that the possibility of a transformation of PIP to sarcoma has been described [30,31], as well as the occurrence of aggressive forms with unfavorable outcome [32,33]. It is likely that PIP includes a complex of diseases ranging from benign fibroistiocitoma to malignant forms, thus explaining the large clinical outcome variability reported in the literature [10].

## Conclusions

Inflammatory pseudotumor of the lung is a rare disease, histologically characterized by the presence of myofibroblasts and chronic inflammatory cells, such as plasma cells, lymphocytes and histiocytes. Whenever possible surgical resection represents the treatment of choice. Major resections are sometimes needed due to the tumor size or local invasiveness. Complete resection is advocated to prevent recurrence. Long term follow-up is needed.

## Consent

Written informed consent was obtained from patients for publication of this report and accompanying images. A copy of the written consent is available for review by the Editor in chief of this journal.

## Competing interests

The authors declare that they have no competing interests.

## Authors' contributions

All authors: 1. have made substantial contributions to conception and design, or acquisition of data, or analysis and interpretation of data; 2. have been involved in drafting the manuscript or revisiting it critically for important intellectual content; 3. have given final approval of the version to be published.
